# Noninvasive ventilation in hypercapnic chronic obstructive pulmonary disease

**DOI:** 10.1186/s13054-017-1854-3

**Published:** 2017-10-26

**Authors:** Silvio A. Ñamendys-Silva

**Affiliations:** 1 Department of Critical Care Medicine, Fundación Clínica Médica Sur, Mexico City, Mexico; 2 Department of Critical Care Medicine, Instituto Nacional de Cancerología, Mexico City, Mexico; 3 Department of Critical Care Medicine, Instituto Nacional de Ciencias Médicas y Nutrición Salvador Zubirán, Mexico City, Mexico; 4 Puente de Piedra 150,Toriello Guerra, Torre 1, Piso 1, 14050 Mexico City, Mexico

Recently, Murphy and colleagues [1] reported findings from a clinical trial designed to evaluate the effect of home noninvasive ventilation (NIV) with oxygen on time to readmission or death in patients with persistent hypercapnia after an acute chronic obstructive pulmonary disease (COPD) exacerbation. The authors concluded that the addition of home NIV to home oxygen therapy may improve outcomes in patients with severe COPD and persistent hypercapnia following hospital admission.

I would like to make the following observations in relation to the use of NIV in patients with COPD:

1. In COPD patients, NIV may be beneficial when used overnight or during the day at home [2]. When using pressure-targeted ventilation, beginning in the spontaneously triggered mode with a backup rate is recommended. The pressure limit should be set to 8 to 12 cm H_2_O inspiratory and 3 to 5 cm H_2_O expiratory, and the inspiratory pressure (10 to 20 cm H2O) should be gradually increased, as tolerated, in order to alleviate dyspnea, decrease respiratory rate, increase tidal volume (if being monitored), and promote good patient–ventilator synchrony [3].
2. The transdiaphragmatic pressure (Pdi; calculated as the difference between the gastric and esophageal pressures), ranged from 10.87 to 14.95 cm H_2_O during spontaneous inspirations. The Pdi represents the gastroesophageal pressure gradient, and it can be considered a driving force for the gastric content to reflux into the esophagus [4]. There is a strong linear correlation between the lower esophageal sphincter (LES) pressure and Pdi. Inspirations made with a closed mouth and nose or a closed glottis (effort levels of 75 to100 %) can cause an increase in the LES pressure of between 127.8 and 179.5 cmH_2_O [4]. NIV may have some problems related to air pressure and flow, such as gastric insufflation (30 to 40%) and aspiration (5%) [5]. Murphy and colleagues [1] did not report on the side effects of the high pressure strategy.
3. One of the long-term objectives of NIV is to prolong survival [3]. However, Murphy and colleagues [1] reported that 12-month mortality results were not different between the groups (28.1% in the home oxygen plus home NIV group and 32.2% in home oxygen alone group [difference of 4.13%, *p* = 0.777]) [1].
4. Finally, the estimated cost of providing a domiciliary NIV service is $3022 in the first year and $1956 in subsequent years [2], which is a high cost for those with low and lower middle incomes.

## Abbreviations

COPD: Chronic obstructive pulmonary disease
LES: Lower esophageal sphincter
NIV: Noninvasive ventilation
Pdi: Transdiaphragmatic pressure
